# Pharmacological activation of PPARβ/δ preserves mitochondrial respiratory function in ischemia/reperfusion *via* stimulation of fatty acid oxidation-linked respiration and PGC-1α/NRF-1 signaling

**DOI:** 10.3389/fendo.2022.941822

**Published:** 2022-08-15

**Authors:** Ioanna Papatheodorou, Marina Makrecka-Kuka, Janis Kuka, Edgars Liepinsh, Maija Dambrova, Antigone Lazou

**Affiliations:** ^1^ Laboratory of Animal Physiology, Department of Zoology, School of Biology, Aristotle University of Thessaloniki, Thessaloniki, Greece; ^2^ Laboratory of Pharmaceutical Pharmacology, Latvian Institute of Organic Synthesis, Riga, Latvia; ^3^ Faculty of Pharmacy, Riga Stradins University, Riga, Latvia

**Keywords:** ischemia/reperfusion, cardioprotection, electron transport system, fatty acid oxidation, mitochondrial respiration, ROS, PPARβ/δ

## Abstract

Myocardial ischemia/reperfusion (I/R) injury leads to significant impairment of cardiac function and remains the leading cause of morbidity and mortality worldwide. Activation of peroxisome proliferator-activated receptor β/δ (PPARβ/δ) confers cardioprotection *via* pleiotropic effects including antioxidant and anti-inflammatory actions; however, the underlying mechanisms are not yet fully elucidated. The aim of this study was to investigate the effect of PPARβ/δ activation on myocardial mitochondrial respiratory function and link this effect with cardioprotection after ischemia/reperfusion (I/R). For this purpose, rats were treated with the PPARβ/δ agonist GW0742 and/or antagonist GSK0660 *in vivo*. Mitochondrial respiration and ROS production rates were determined using high-resolution fluororespirometry. Activation of PPARβ/δ did not alter mitochondrial respiratory function in the healthy heart, however, inhibition of PPARβ/δ reduced fatty acid oxidation (FAO) and complex II-linked mitochondrial respiration and shifted the substrate dependence away from succinate-related energy production and towards NADH. Activation of PPARβ/δ reduced mitochondrial stress during *in vitro* anoxia/reoxygenation. Furthermore, it preserved FAO-dependent mitochondrial respiration and lowered ROS production at oxidative phosphorylation (OXPHOS)-dependent state during ex vivo I/R. PPARβ/δ activation was also followed by increased mRNA expression of components of FAO -linked respiration and of transcription factors governing mitochondrial homeostasis (carnitine palmitoyl transferase 1b and 2-CPT-1b and CPT-2, electron transfer flavoprotein dehydrogenase -ETFDH, peroxisome proliferator-activated receptor gamma co-activator 1 alpha- PGC-1α and nuclear respiratory factor 1-NRF-1). In conclusion, activation of PPARβ/δ stimulated both FAO-linked respiration and PGC-1α/NRF -1 signaling and preserved mitochondrial respiratory function during I/R. These effects are associated with reduced infarct size.

## Introduction

Acute myocardial infarction (MI) and the heart failure that often follows, remain the leading cause of mortality and morbidity worldwide. Timely myocardial reperfusion, which remains the only treatment of choice up to date, can paradoxically exacerbate myocardial injury and cardiomyocyte death, known as ischemia/reperfusion (I/R) injury (1). Despite intense research efforts to prevent the development of I/R injury and heart failure and reduce the size of myocardial infarction, translation of cardioprotection to clinical practice has proven difficult. Potential reasons may include the complexity of mechanisms underlying I/R or the lack of rigor and/or reproducibility of preclinical studies (2). In this respect, practical guidelines aim to improve the likelihood of translating novel cardioprotective interventions into the clinical setting for patient benefit (3–5).

Mitochondrial dysfunction is a key determinant of I/R injury (6–8). Under normal conditions, mitochondrial respiration depends on electron flow through the four protein complexes of the inner mitochondrial membrane that comprise the electron transport system (ETS), creating a membrane potential that is essential for energy production (9). ETS activity is tightly regulated by the nuclear respiratory factors (NRFs) and the peroxisome proliferator activated receptor γ co-activator 1α (PGC-1α). However, ischemia brings mitochondrial oxidative phosphorylation (OXPHOS) to a halt leading to ATP deficiency, malfunction of ETS, collapse of the inner membrane potential and eventually mitochondrial dysfunction and cell death (6). Importantly, restoration of oxidative metabolism at the offset of reperfusion creates a burst of intramitochondrial ROS possibly because rapid changes in energy metabolism and oxygen levels overwhelm the ETS with electrons (10). Therefore, approaches that aim to alleviate or diminish mitochondrial damage during I/R are promising cardioprotective strategies (8, 11).

Peroxisome proliferator-activated receptors (PPAR α, β/δ and γ) are critical transcriptional regulators of myocardial lipid and energy metabolism (12), which exert anti-inflammatory, antioxidant and cardioprotective effects (13–16). PPARβ/δ isoform is expressed in most tissues with particular abundance in those with high level of FA oxidation (FAO) including heart, liver, kidney, skeletal muscle and pancreas (17, 18). PPARβ/δ is the predominant form in cardiac cells. PPARβ/δ deficiency in the adult heart impairs expression of antioxidant enzymes and transcriptional regulators of mitochondrial biogenesis leading to cardiac hypertrophy and dysfunction (19), while a constitutively active PPARβ/δ upregulates myocardial oxidative metabolism and enhances mitochondrial biogenesis (20). Pharmacological activation of PPARβ/δ protects cardiomyocytes against oxidative stress through suppression of ROS generation and downregulation of pro-apoptotic factors (21) and decreases infarct size after ex vivo ischemia/reperfusion (I/R) (22–24). In particular, we have recently shown that stimulation of mitochondrial function and preservation of ATP production are involved in PPARβ/δ -mediated cardioprotection in I/R (24). However, the role of PPARβ/δ on cardiac mitochondrial respiratory function during I/R is yet to be fully elucidated.

In the present study, we sought to investigate the role of PPARβ/δ as potential modulator of myocardial mitochondrial respiration and ROS production in I/R. We employed a PPARβ/δ specific agonist to activate the receptor and an experimental model of ex vivo perfused heart subjected to I/R to determine infarct size as a robust endpoint of cardioprotection (3, 5). In addition to measuring mitochondrial respiratory function, we sought to also elucidate the underlying molecular mechanisms of PPARβ/δ-mediated effects focusing on PGC-1α/NRF-1 signaling axis, which regulates mitochondrial homeostasis.

## Materials and methods

### Animals

All experimental procedures were performed on male Wistar rats (250–350g body weight); all efforts were made to minimize suffering. Animals were obtained from Laboratory Animal Centre, University of Tartu (Tartu, Estonia). Animals were left to acclimate for two weeks prior to the experiments. All animals were housed under constant temperature (20–23°C) and relative humidity (45–65%) with a 12h light/dark cycle and with unlimited access to food (R70 diet, Lactamin AB, Kimstad, Sweden) and water. All protocols and procedures were performed at the Latvian Institute of Organic Synthesis in Riga, Latvia according to the guidelines of the European Commission and the ARRIVE guidelines (25, 26). The procedures were approved by the Latvian Animal Protection Ethical Committee of the Food and Veterinary Service, Riga, Latvia.

### Animal groups and compound administration protocol

Animals were randomly divided into four groups according to treatment. The agonist GW0742 and the antagonist GSK0660 were diluted in DMSO. Control group animals were given intraperitoneal (IP) bolus of 0.5 mL/kg of 10% DMSO. GW group animals were given 1mg/kg (IP) of the specific PPARβ/δ agonist, GW0742 (CAS 317318-84-6, Santa Cruz Biotech., Dallas, Texas, USA) 24 h before experimental procedures. GSK/GW animals received 3mg/kg (i.p) of PPARβ/δ antagonist GSK0660 (CAS 1014691-61-2, Santa Cruz Biotech., Dallas, Texas, USA) and then 1mg/kg (IP) of GW0742 at 30 h and 24 h, respectively, before experimental procedures. Dosage for the agonist and the antagonist was based on preliminary experiments and previously published studies (24). Antagonist GSK0660 was administered before GW0742 with the aim to verify that any observed findings were mediated by PPARβ/δ activation. For studies involving high resolution fluorespirometry and molecular analysis, 3-5 animals per experimental group were used. For infarct size determination, the number of animals used was 5-8 per experimental group. Heart tissues were collected and processed for high-resolution fluorespirometry or were cannulated *via* the aorta and processed for ex vivo I/R. Experimental procedures were performed in a blinded way.

### *Ex vivo* myocardial I/R

Animals were anesthetized by administration of sodium pentobarbital (60 mg/kg ip), and heparin (1000 IU/kg) was administered concomitantly. Hearts were cannulated *via* the aorta and retrogradely perfused at Langendorff apparatus at a constant perfusion pressure of 60 mmHg at 37°C. Perfusion buffer was a Krebs-Henseleit buffer (118mM NaCl, 4.7mM KCl, 2.52mM CaCl2, 1.64mM MgCl2, 24.88mM NaHCO3, 1.18mM KH2PO4 and 0.05mM EDTA, pH=7.4), oxygenated with 95% O_2_ - 5% CO_2_. Hearts were left to stabilize for 20 minutes before induction of regional I/R. Left anterior descending coronary artery (LAD) was occluded by binding the vessel with a 4-0 suture, for 30 minutes. Reperfusion was initiated by liberation of LAD and continued for 60 minutes for hearts that were used for high resolution fluorespirometry and molecular analysis. The risk area (RA) and the non-risk area (NRA) of those hearts were separated at the end of reperfusion and were either processed for homogenate preparation or were stored at -80°C for further molecular analysis. Hearts that were used for infarct size determination were reperfused for 2 hours.

### Preparation of cardiac tissue homogenate and intact cardiac mitochondria

Cardiac tissue homogenate and cardiac subsarcolemmal mitochondria were prepared as previously described (27) and were used for high-resolution fluorespirometry according to the requirements of each protocol. In brief, cardiac tissues were weighed and then minced and homogenized in 2mL of isolation buffer A (180mM KCl, 10mM Tris-HCl, and 1mM EGTA, pH 7.4 at 4°C) in a glass Potter homogenizer. The homogenate was centrifuged for 5 min at 750×g, 4°C. The supernatant containing intact cardiac mitochondria (mitochondria-containing homogenate) was preserved on ice and was used for the high resolution fluorespirometry anoxia/reoxygenation protocol and the determination of mitochondrial function in the I/R-subjected hearts. For the isolation of subsarcolemmal mitochondria, the heart tissue homogenate was centrifuged at 6800×g for 10 min, at 4°C. The mitochondrial pellet was resuspended in isolation buffer B (180mM KCl, 20mM TrisCl, pH=7.2), centrifuged at 6800×g for 10 min, at 4°C and then resuspended in 200μl of isolation buffer B. Intact mitochondria were used for determination of fatty acid oxidation-dependent mitochondrial function in the healthy heart. Protein concentration was determined spectrophotometrically by the Lowry method (28) using BSA as a standard.

### High-resolution fluorespirometry

Mitochondrial respiration and ROS production rates were determined at 37°C using Oxygraph 2k systems (O2k; Oroboros Instruments, Innsbruck, Austria) in MiR05 media (110 mM sucrose, 0.5 mM EGTA, 3 mM MgCl2, 10 mM KH2PO4, 20 mM taurine, 60 mM K-lactobionate, 20 mM HEPES, pH 7.1, 0.1% BSA essentially free of fatty acids) as previously described (27). Briefly, palmitoyl carnitine was added at 10μM in combination with malate (0.5 mM, to prevent oxaloacetate -mediated blockage of Krebs cycle) to determine FAO‐dependent mitochondrial respiration (FADH2 (NADH)‐pathway; F(N)‐ pathway) at LEAKn, the substrate dependent state. ADP (D) was added at 5mM to stimulate oxidative phosphorylation (OXPHOS)‐dependent respiration (OXPHOS state). Pyruvate (P) (5mμ, complex I substrate, N‐pathway) was used to activate FN‐pathway linked respiration and to measure pyruvate metabolism supported respiration in the presence of FAO substrates. Succinate (S) (10mM, complex II substrate, S‐pathway) was added to reconstitute convergent FNS-linked respiration. Since complexes I and II constitute the electron entry points of the ETS, successive stimulation of complexes I and II activates all three F, N and S-linked pathways and leads to progressive stimulation of the full ETS -OXPHOS capacity. Rotenone (Rot) (0.5µM, inhibitor of complex I) was added to determine S-linked respiration (5, 21). To simulate I/R *in vitro*, cardiac homogenate was subjected to anoxia/reoxygenation (Anox/Reox) in O2k oxygraph systems in MIR05 media. Initially, mitochondrial respiration and ROS production rates were determined in the presence of 10mμ S (S-pathway) and 0.5μμ of Rot to simulate succinate accumulation and Complex I dysfunction that occurs under ischemic conditions. Then 5mμ of ADP was added to initiate OXPHOS state and oxygen levels were left to drop completely. The cardiac homogenate was subjected to 30 minutes of anoxia followed by 10 minutes of reoxygenation. Post-Anox/Reox values for mitochondrial respiration and ROS production rates, as well as H_2_O_2_/O ratio, were expressed as % of the respective pre-anoxic values.

In both described protocols ROS production was determined by measurement of the H_2_O_2_ flux simultaneously with oxygen consumption in each O2k chamber. Measurements were done by addition of 10μM of Amplex^®^ UltraRed (AmR), H_2_O_2_-sensitive probe, 1U/mL of horseradish peroxidase (HRP) and 5U/mL of superoxide dismutase (SOD). The reaction product between AmR and H_2_O_2_ is catalyzed by HRP to produce the red-fluorescent oxidation product, resorufin (29, 30). Calibrations of the AmR signal were performed with H_2_O_2_ titrations of 0.1µM at specific timepoints during the measurement. Oxygen consumption and ROS production rates were registered by the DatLab software (OROBOROS 150 INSTRUMENTS, Innsbruck, Austria) according to the oxygen and fluorescent signal over time. The percentage of superoxide production per atom of oxygen (H_2_O_2_/O, %) was calculated. Non-mitochondrial respiration was used as a correction factor for oxygen consumption and was measured by the addition of 2.5μM Antimycin A (AA). All O2k substrates used in respirometry were from Sigma (St. 154 Louis, Missouri, US). To determine the contribution of each pathway to the respiration rate, respective flux control factors (FCF) were calculated with the following formula: FCF = (respiration rate after the addition of substrate - respiration rate before the addition of substrate)/respiration rate after the addition of substrate.

### Determination of infarct size

Infarct size was determined as described previously (31, 32). Briefly, LAD was re‐occluded at the end of reperfusion and the heart was perfused with 0.1% methylene blue dissolved in perfusion buffer. Cardiac ventricles were transversely cut into 2mm thick slices and treated with 1% triphenyl‐tetrazolium chloride (TTC) in 0.1M sodium phosphate buffer for 10mins at 37°C. Slices were photographed and infarct size was measured by planimetry using Image‐Pro Plus v6.3 software (Media Cybernetics Inc., Rockville, MD, USA) in order to determine the risk area (RA) and the necrotic area (NA) of the heart. The obtained values were then used to calculate the infarct size (IS) as % percentage of the RA, according to the formula IS = NA/RA × 100%.

### Isolation of total RNA and real-time PCR

Total RNA from frozen cardiac tissue was obtained using the TRI-based ExtraZOL reagent (Blirt S.A., Gdansk, Poland) according to the manufacturer’s protocol. After isolation, RNA samples were resuspended in 20μl of sterile water and were quantified using a Quawell Q5000 micro-volume UV-Vis spectrophotometer at 260nm. Reverse transcription was performed on 1μg of isolated RNA using PrimeScript RT Reagent Kit (Takara BIO, Kusatsu, Japan) in a 20 μL reaction volume as previously described (21). Real-Time PCR analysis was performed by addition of 0.025μg of cDNA and 10pmol of the desired forward and reverse primers to the SYBR Green Master Mix (KAPA Biosystems, Wilmington, MA, USA) according to the manufacturer’s protocol. Experiments were run on an Applied Biosystems 7500 instrument (Applied Biosystems, Waltham, MA, USA) and relative mRNA expression was normalized to β-actin. Relative changes in expression were calculated using the 2^(-ΔΔCt)^ method. Primers for the examined genes are given in **Table 1**.

**Table 1:** Primer sequences used for gene amplification .

Gene	Forward Primer	Reverse Primer
*Angptl4*	GACTTTTCCAGATCCAGCCT	TGATTGAAGTCCACAGAGCC
*Cpt-1b*	TCTGGGCTTCTGTGTTCGTC	AATTGTGGCTGGCACACTG
*Cpt-2*	GCTCCGAGGCATTTGTC	CATCGCTGCTTCTTTGGT
*Etfdh*	GAGCAGTAGGGGCTTCTTGG	TGATATGCAGGGCAGGACAG
*Nrf1*	CATGGAGGAGCACGGAGTGA	AGCAGCCAGATGGGCAGTTA
*Ppargc-1α*	AGAGTCACCAAATGACCCCA	GAGTTAGGCCTGCAGTTCCA
*Sdha*	CGGTATGCCGTTTAGCAGGA	GTGTCATACCGCAGAGATCGT

### Protein extraction and immunoblotting

Cardiac tissue was homogenized in ice-cold lysis buffer containing 20mM β-glycerophosphate, 50mM NaF, 2mM EDTA, 10mM benzamidine, 20mM Hepes, 0.2mM Na_3_VO_4_, 5mM dithiothreitol (DTT), protease inhibitors (0.2mM leupeptin, 0.3 mM phenyl methyl-sulphonyl fluoride (PMSF), 0.12mM pepstatin, 0.01mM trans-epoxy succinyl L-leucylamido-(4-guanidino) butane (E64) and 1% (w/v) Triton X-100. Samples were left to extract on ice for 30 minutes and were then centrifuged at 11,000×g for 15 min at 4°C. Total protein concentration was determined spectrophotometrically using the Biorad assay (BioRad, Hercules, CA, USA). One-third volume of SDS-PAGE sample buffer containing 10% SDS (w/v), 13% glycerol (v/v), 300 mM Tris–HCl pH 6.8, 130 mM DTT and 0.2% bromophenol blue (w/v). Samples migrated on 10% poly-acrylamide gels containing 375mM Tris-HCl pH 8.8, 0.275% (w/v) bis-acrylamide, 0.1% (v/v) SDS, 0.15% (w/v) ammonium persulfate and 0.007% (v/v) TEMED at a constant voltage of 120V. Proteins were then transferred to a 0.45mm pore Amersham™Protran^®^ nitrocellulose membrane (Merck KGaA, Darmstadt, Germany) using a stable voltage of 12V for 1h. Non-specific binding sites were blocked with 5% (w/v) non-fat milk powder in TBST buffer (20mM Tris–HCl pH 7.6, 137mM NaCl, and 0.1% (v/v) Tween 20) for 30 min at room temperature. Membranes were incubated overnight at 4°C with primary antibodies diluted according to the manufacturers’ instructions in TBST buffer containing 5% (w/v) bovine serum albumin (BSA), and then washed in TBST buffer. Membranes were incubated for 60 min at room temperature with HRP-conjugated secondary antibodies in TBST buffer containing 5% (w/v) BSA. After washing with TBST, proteins of interest were detected by enhanced chemiluminescence (Signal Fire ECL reagent - Cell Signaling, Beverly, MA, USA) and were quantified by scanning densitometry. The primary antibodies used were anti-PPARβ/δ (#101720, Cayman Chemical, Ann Arbor, Michigan, USA), anti-CPT-2 (#PA5-12217, Thermo Fisher Scientific, Waltham, Massachusetts, USA), anti-PGC-1α (sc-13067, Santa Cruz Biotech., Dallas, Texas, USA) and anti-SDHA (#ab14715, Abcam, Cambridge, UK). The secondary antibodies were anti-mouse IgG (#P0447, Dako, Glostrup Municipality, Glostrup, Denmark) and anti-rabbit IgG (#7074, Cell Signaling, Beverly, MA, USA).

### Statistical analysis of data

Data are presented as the mean ± S.E.M. Statistical significance between the two groups was evaluated with an unpaired t test. Statistically significant differences in the mean values of three or more groups were evaluated using one-way ANOVA, followed by Dunnett T3 posttest. Statistical significance was established at p<0.05.

## Results

### PPARβ/δ is essential for basal mitochondrial respiration rate

**Figure 1:**
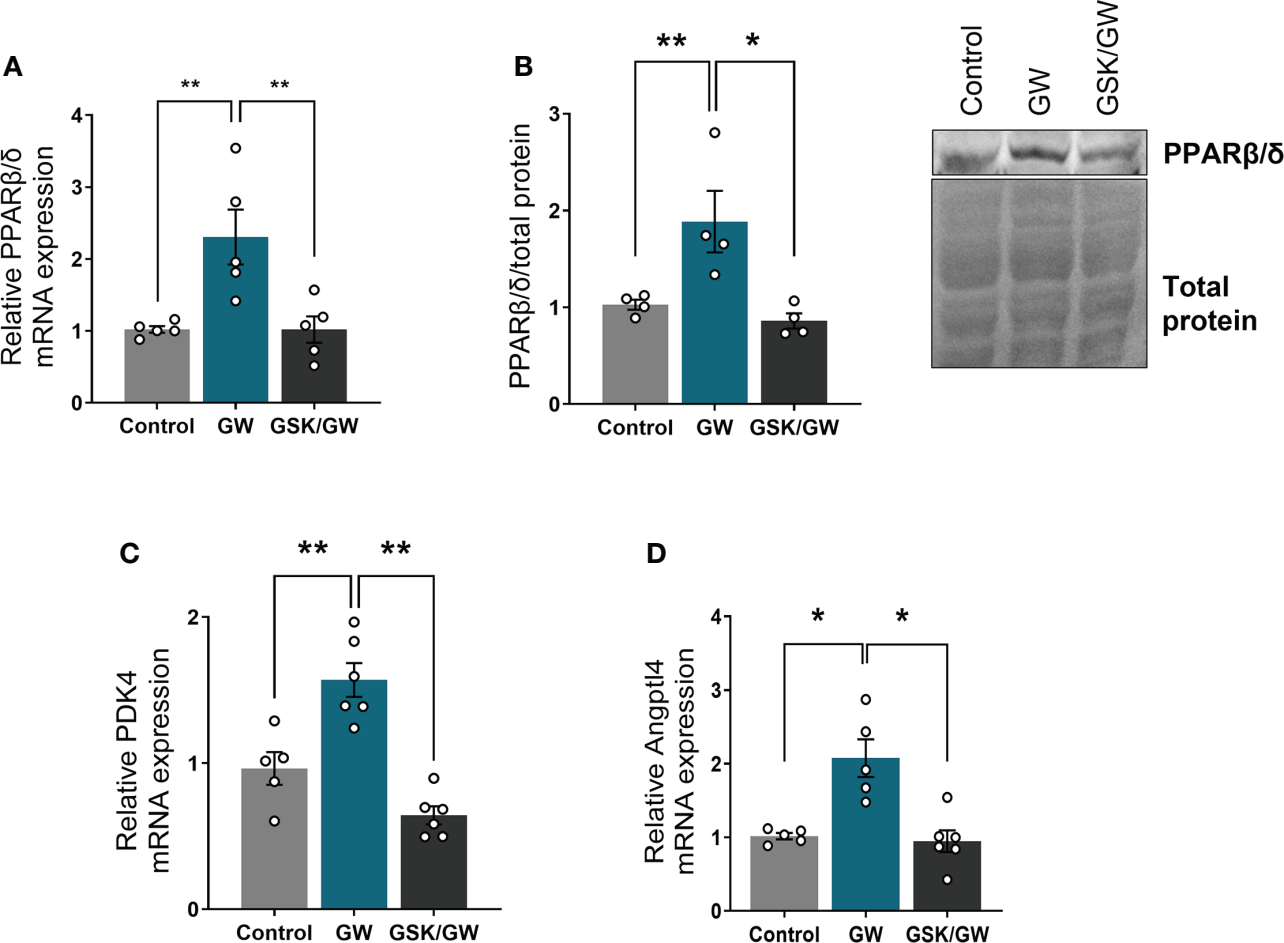
PPARβ/δ is activated after administration of GW0742. **(A)** mRNA expression of PPARβ/δ was quantified by qRT-PCR and normalized to β-actin. Results are presented as mean ± SEM of 5-6 independent experiments, **(B)** Representative immunoblot of PPARβ/δ protein (upper panel) and quantification by densitometric analysis (lower panel). Results are presented as mean ± SEM of 4-5 independent experiments, **(C)** mRNA expression of PPARβ/δ target gene PDK4 and **(D)** Angptl4 were quantified by qRT-PCR and normalized to β-actin. Results are presented as mean ± SEM of 5-6 independent experiments. *p < 0.05, **p < 0.01.

To confirm activation of PPARβ/δ in the heart, when agonist GW0742 was administered, mRNA expression and protein levels of PPARβ/δ, as well as mRNA expression of two established PPARβ/δ downstream targets, pyruvate dehydrogenase kinase 4 (PDK4) (33) and angiopoietin like peptide 4 (Angptl4) (34), were determined. Then, in order to verify that any effects are mediated by PPARβ/δ, the antagonist GSK0660 was administered before the agonist. Administration of GW0742 led to an almost two-fold increase both in the mRNA and protein levels of PPARβ/δ compared with the Control group, while administration of GSK0660 prior to GW0742 abolished this effect (**Figures 1A, B**). Similarly, administration of GW0742 resulted in increased mRNA levels of PDK4 (**Figure 1C**) and Angptl4 by almost twofold compared with the Control group hearts (**Figure 1D**). The above results demonstrate effective PPARβ/δ activation following GW0742 administration. Administration of the PPARβ/δ antagonist GSK0660 prior to GW0742, abolished the upregulation of PPARβ/δ downstream targets and brought both PDK4 and Angptl4 mRNA expression back to the Control level, demonstrating effective attenuation of PPARβ/δ activation (**Figures 1C, D**).

**Figure 2:**
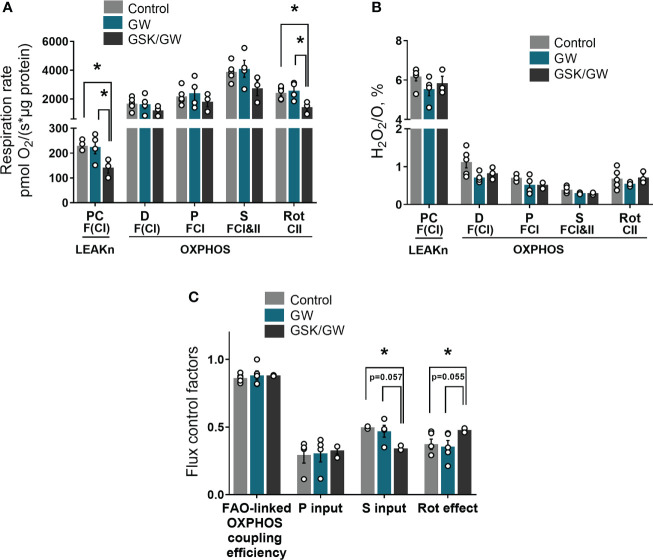
PPARβ/δ inhibition modulates basal mitochondrial respiration rate. **(A)** Respiration rate in intact cardiac mitochondria **(B)** H_2_O_2_ % ratio between O_2_ flow and H_2_O_2_ production rate, per μg of protein in the presence of PC (palmitoyl-carnitine), D (ADP), P (pyruvate), S (succinate) and Rot (rotenone – complex I inhibitor). The fluorescence signals were calibrated using H_2_O_2_ titrations at the corresponding state **(C)** Substrate flux control factors calculated as: (respiration rate after the addition of substrate - respiration rate before the addition of substrate)/respiration rate after the addition of substrate. PC, palmitoyl-carnitine; D, ADP; P, pyruvate; S, succinate; Rot, rotenone – complex I inhibitor; F(N), fatty acid oxidation-dependent pathway (FADH2 and (NADH)); S, succinate-dependent pathway (FADH2); LEAKn, substrate metabolism-dependent state; OXPHOS, oxidative phosphorylation-dependent state; FAO, fatty acid oxidation; GW, pretreated with PPARβ/δ agonist GW0742; GSK/GW, pretreated with PPARβ/δ antagonist GSK0660 and then pretreated with PPARβ/δ agonist GW0742. Results are presented as means ± SEM of 3-5 independent experiments. *p < 0.05.

Considering that PPARβ/δ regulates FAO in the heart (18), we then examined the effect of PPARβ/δ activation on FAO-dependent mitochondrial function in isolated cardiac mitochondria *in vitro.* For this purpose, FAO-dependent respiration and ROS production rates were determined in LEAKn and OXPHOS states in isolated cardiac mitochondria. While activation of PPARβ/δ did not affect mitochondrial respiration, inhibition of PPARβ/δ with GSK0660 (GSK/GW group) resulted in a significant reduction of mitochondrial F(CI)-linked respiration in LEAKn state, compared with Control or GW group (**Figure 2A**). CII-linked respiration rate of GSK/GW group was also significantly lower than Control or GW group in the OXPHOS state (**Figure 2A**). However, no differences were observed in ROS production, expressed as H_2_O_2_/O ratio, in the presence of GW0742 or GSK0660/GW0742 in all studied states (**Figure 2B**). Furthermore, inhibition of PPARβ/δ resulted in decreased succinate input and increased Rotenone effect, calculated as FCF, in GSK/GW group compared with the Control group, indicating a metabolic shift away from the S pathway and towards the NADH pathway (**Figure 2C**). On the contrary, no effect was observed in pyruvate input or FAO-linked OXPHOS coupling efficiency. Taken together these findings suggest that, while PPARβ/δ activation does not affect mitochondrial functionality under normal conditions, the activity of the receptor is essential for basal F- and/or CII- linked mitochondrial metabolism.

### PPARβ/δ activation reduces ROS production after *in vitro* anoxia/reoxygenation

**Figure 3:**
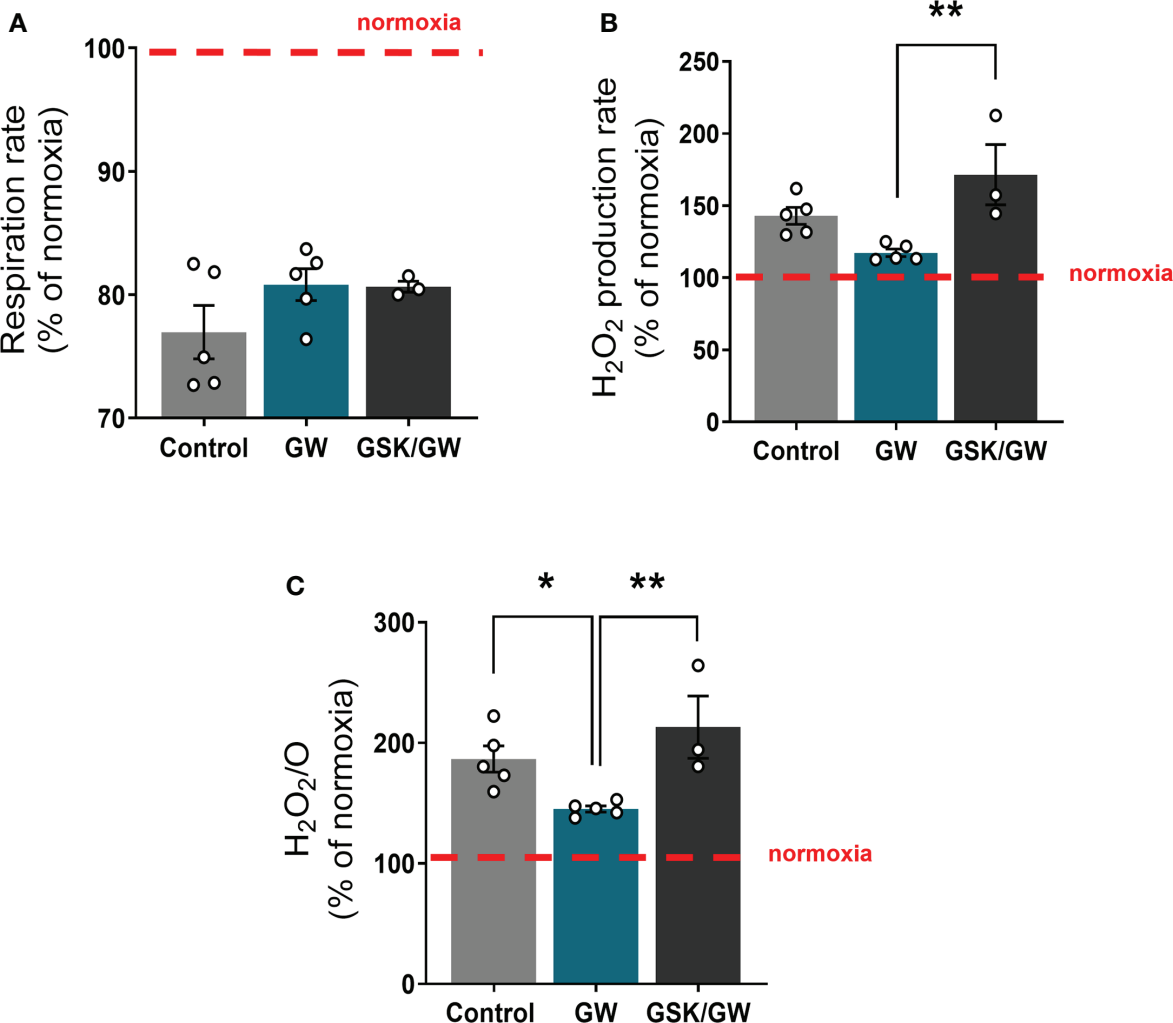
PPARβ/δ activation ameliorates mitochondrial ROS production after *in vitro* anoxia/reoxygenation. **(A)** Respiration rate in cardiac homogenate **(B)** H_2_O_2_ production rate corrected for the background slope determined in the absence of homogenate **(C)** H_2_O_2_ % ratio between O_2_ flow and H_2_O_2_ production rate, all calculated as the ratio between the post-anoxia/reoxygenation values divided by the respective normoxic values. The fluorescence signals were calibrated using H_2_O_2_ titrations at the corresponding state. Results are presented as means ± SEM from 3-5 independent experiments. GW, pretreated with PPARβ/δ agonist GW0742; GSK/GW, pretreated with PPARβ/δ antagonist GSK0660 and then pretreated with PPARβ/δ agonist GW0742. *p < 0.05, **p < 0.01.

To determine whether activation of PPARβ/δ affects mitochondrial functionality under stress conditions, mitochondria-containing cardiac homogenate was subjected to anoxia/reoxygenation (Anox/Reox). Post-Anox/Reox mitochondrial respiration and ROS production rates, which are directly associated with mitochondrial functionality, as well as H_2_O_2_/O ratio, were determined and expressed as % of the respective normoxic values. While activation of PPARβ/δ did not affect mitochondrial respiration rate (**Figure 3A**), the Anox/Reox-induced increase in ROS production rate tended to be reduced (**Figure 3B**) and H_2_O_2_/O ratio was significantly reduced by 22% as compared with the Control group (**Figure 3C**). On the other hand, prevention of PPARβ/δ activation by the addition of the antagonist GSK0660 (GSK/GW group), led to an increase in both ROS production rate and H_2_O_2_/O ratio, in comparison to the GW group (**Figures 3B, C**). These results indicate a ROS-reducing effect of PPARβ/δ activation on mitochondria-containing cardiac homogenate during *in vitro* Anox/Reox.

### PPARβ/δ activation preserves FAO-dependent mitochondrial respiratory function and reduces infarct size in I/R

**Figure 4:**
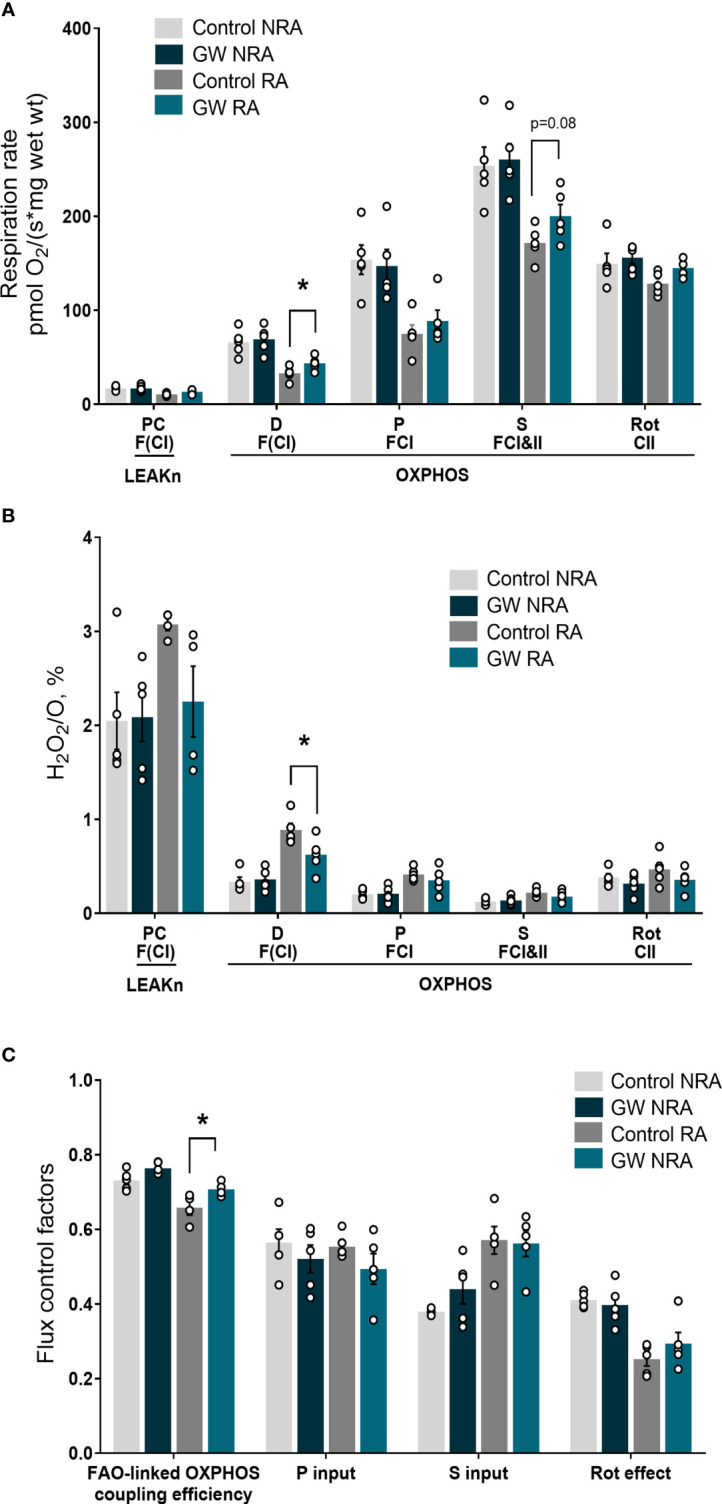
PPARβ/δ activation improves FAO-dependent mitochondrial bioenergetics in *ex vivo* I/R. **(A)** Respiration rate **(B)** H_2_O_2_ % ratio between O_2_ flow and H_2_O_2_ production rate. Fluorescence signals were calibrated using H_2_O_2_ titrations at the corresponding state. Data is presented as means ± SEM of 4-6 independent experiments. **(C)** Substrate flux control factors calculated as: (respiration rate after the addition of substrate - respiration rate before the addition of substrate)/respiration rate after the addition of substrate. PC, palmitoyl carnitine; D, ADP; P, pyruvate; S, succinate; Rot, rotenone – complex I inhibitor; RA, risk area; NRA, non-risk area; FAO, fatty acid oxidation; F(N), fatty acid oxidation-dependent pathway (FADH2 and NADH); S, succinate-dependent pathway (FADH2); LEAKn, substrate metabolism-dependent state; OXPHOS, oxidative phosphorylation-dependent state; GW, pretreated with PPARβ/δ agonist. *p < 0.05.

**Figure 5:**
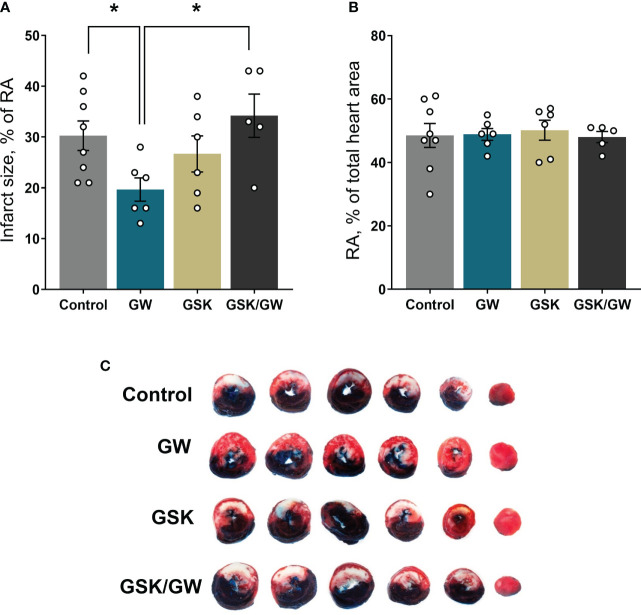
Activation of PPARβ/δ leads to reduced infarct size after *ex vivo* I/R. **(A)** NA (necrotic area) size as % percentage of the RA (risk area) size after *ex vivo* LAD-occlusion and reperfusion (*ex vivo* I/R) **(B)** % of the RA size compared with total cardiac tissue area. **(C)** Representative images of the stained cardiac slices for each experimental group. Infarct size was determined with TTC staining and measured using imaging software. Results are presented as means ± SEM from 5-8 independent experiments. RA, risk area; NA, necrotic area; LAD, left anterior descending coronary artery; GW, pretreated with PPARβ/δ agonist GW0742; GSK/GW, pretreated with PPARβ/δ antagonist GSK0660 and then pretreated with PPARβ/δ agonist GW0742. *p < 0.05.

Mitochondrial dysfunction plays a key role in driving oxidative stress and I/R injury (4). To corroborate the beneficial effect of PPARβ/δ on mitochondrial respiration and ROS generation in a more physiologically relevant model, we examined the effect of PPARβ/δ on FAO-dependent mitochondrial respiration rate and ROS production in mitochondria-containing homogenate from *ex vivo* isolated heart preparations subjected to regional I/R. Activation of PPARβ/δ resulted in a 24% increase in F(CI)-linked mitochondrial respiration rate and a 30% decrease in H_2_O_2_/O ratio at the OXPHOS state, in the risk area (RA) (**Figures 4A, B**) suggesting increased OXPHOS efficiency. In order to corroborate this notion, FAO-dependent OXPHOS coupling efficiency was calculated for the risk area (RA) and non-risk area (NRA). PPARβ/δ activation was accompanied by a significant increase in OXPHOS coupling efficiency of the RA (**Figure 4C**). The above effects were not evident in the NRA. Furthermore, activation of PPARβ/δ was accompanied by an approximately 30% decrease in the necrotic area/risk area (NA/RA) ratio, as compared with the control (**Figure 5**), an effect that was abolished when PPARβ/δ antagonist GSK0660 was administered before GW0742. PPARβ/δ antagonist GSK0660 alone did not affect infarct size as compared with the control group. Taken together, these results suggest that the infarct size-limiting effect of PPARβ/δ activation during myocardial I/R can be, at least partly, attributed to improved mitochondrial respiratory function after I/R.

### PPARβ/δ activation promotes the upregulation of genes involved in mitochondrial energetic homeostasis

**Figure 6:**
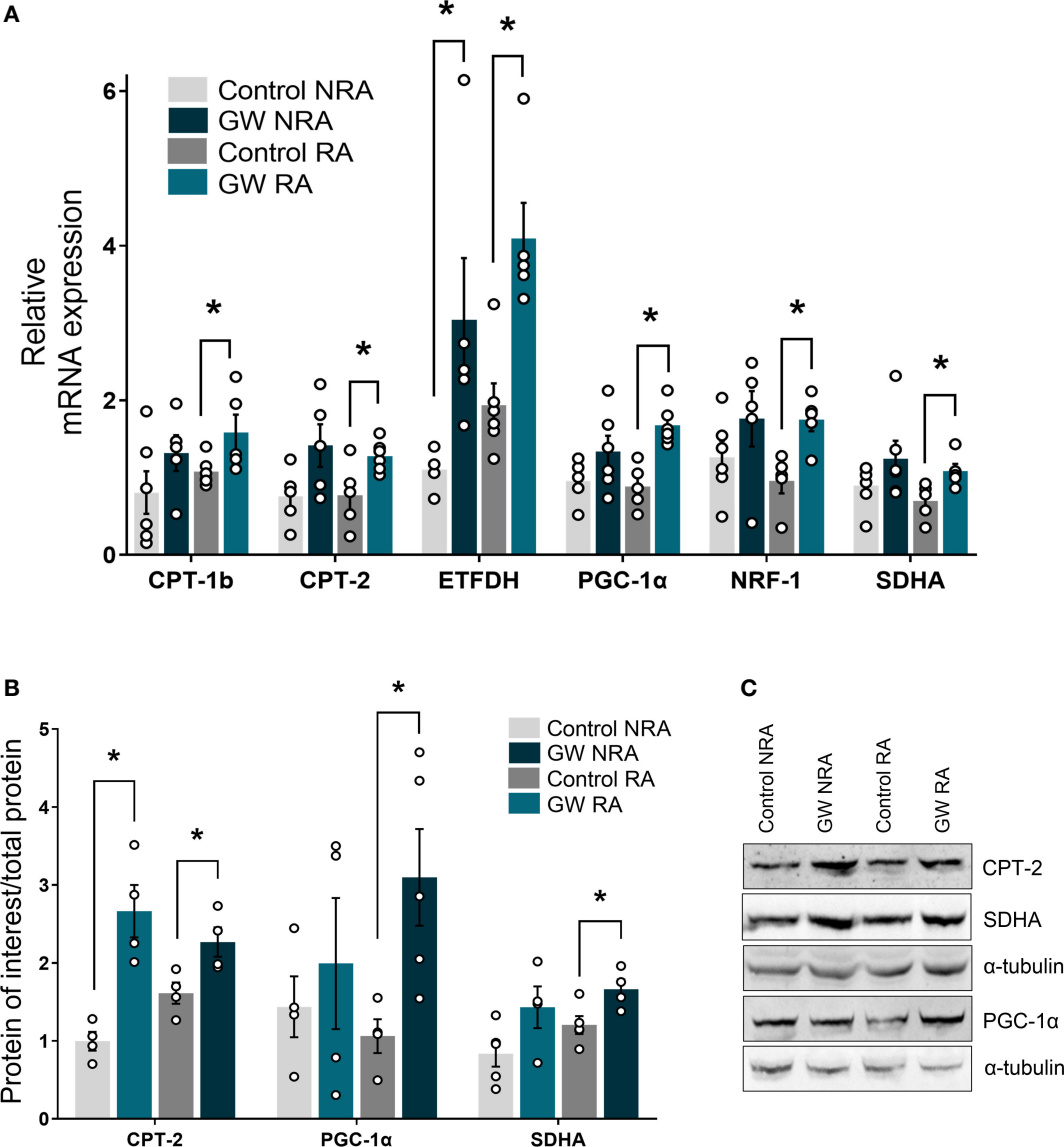
PPARβ/δ upregulates genes of the FAO-dependent electron production and PGC-1α/NRF-1 signaling during I/R. mRNA expression of **(A)** carnitine palmitoyl transferase 1 (CPT-1), carnitine palmitoyl transferase 2 (CPT-2) and electron transfer flavoprotein dehydrogenase (ETFDH), PPAR co-factor α (PGC-1α), nuclear respiratory factor 1 (NRF-1) and succinate dehydrogenase subunit A (SDHA). mRNA expression was determined in the NRA and the RA of *ex vivo* I/R-subjected hearts by qPCR and normalized to β-actin. Results are presented as means ± SEM of 5-6 independent experiments **(B)** CPT-2, PGC-1α and SDHA protein levels quantified by densitometric analysis and **(C)** representative blot images for each protein. Results are presented as means ± SEM of 4-5 independent experiments. RA, risk area; NRA, non-risk area; GW, pretreated with PPARβ/δ agonist GW0742. *p < 0.05.

In an attempt to gain insight into the molecular mechanism underlying the involvement of PPARβ/δ in mitochondrial bioenergetics, the expression of genes implicated in FAO-linked respiration, as well as in mitochondrial respiratory homeostasis in general, was determined. PPARβ/δ activation promoted upregulation of mRNA expression of mitochondrial FA uptake enzymes, carnitine palmitoyltransferase 1b and 2 (CPT-1b, CPT-2), as well as of electron transferring enzyme, electron transfer flavoprotein dehydrogenase (ETFDH), in the risk area, in comparison to the control group (**Figure 6A**). Protein levels of CPT-2 were increased in the RA following PPARβ/δ activation (**Figure 6B**) suggesting overall increased fatty acid substrate channeling towards FAO. Furthermore, PPARβ/δ activation was accompanied by increased mRNA expression of transcription factors peroxisome proliferator-activated receptor gamma co-activator 1 alpha (PGC-1α) and nuclear respiratory factor 1 (NRF-1), which govern mito-nuclear communication, mitochondrial biogenesis and respiratory function (35, 36). In addition, mRNA expression of the NRF-1 downstream target, succinate dehydrogenase A (SDHA) (37) was increased in RA (**Figure 6A**). Protein levels of PGC-1α and SDHA were also increased in RA (**Figures 6C, D**) corroborating the activation of the PGC-1α/NRF-1 axis. These results suggest that stimulation of FA-linked respiration and PGC-1α/NRF-1 signaling are associated with the cardioprotective effects of PPARβ/δ during myocardial I/R.

## Discussion

In this work, we demonstrate that pharmacological activation of nuclear receptor PPARβ/δ enhances mitochondrial FAO-dependent electron flow, stimulates PGC-1α/NRF-1 signaling and preserves mitochondrial respiratory function during myocardial I/R. These results are associated with reduced infarct size.

The role of PPARβ/δ as an essential transcriptional regulator of myocardial FAO and antioxidant defense is well established (12, 16, 24). Furthermore, using inducible gene targeting approaches to inactivate or constitutively activate PPARβ/δ in the adult mice heart, it was shown that PPARβ/δ is required to maintain normal mitochondrial biogenesis and function (19, 20). However, a direct link of PPARβ/δ activation with mitochondrial respiration in the healthy heart as well as in I/R has not been established. The present study demonstrates that PPARβ/δ activation does not affect mitochondrial respiratory function under basal conditions. However, abolishment of PPARβ/δ activation, using the specific antagonist, is accompanied by defective mitochondrial respiration possibly because of impaired FAO-dependent or S pathway-linked mitochondrial respiration, thus pointing to the critical role of PPARβ/δ in maintaining basal mitochondrial respiratory function in the heart. On the other hand, PPARβ/δ activation attenuates ROS production during *in vitro* anoxia/reoxygenation, suggesting a mitoprotective effect of the receptor under conditions of mitochondrial stress. Previous studies have demonstrated pleiotropic beneficial effects of PPARβ/δ activation in cell and animal models of oxidative stress, including downregulation of oxidative stress mediators (23) and pro-apoptotic molecules (22), as well as transcriptional stimulation of the antioxidant defense of the heart (21, 24). Even though these PPARβ/δ-mediated antioxidant effects could result in better mitochondrial function, this is the first time to demonstrate a direct ROS-reducing effect of the receptor on mitochondrial respiratory function. Other studies have associated the activation of PPARβ/δ with the attenuation of inflammatory signaling, such as NF-κβ and IL-6 induced STAT-3 phosphorylation, in cardiac myocytes and liver cells (38, 39). Although, these pathways play an important role in the maladaptive myocardial remodeling after I/R (40), the role of PPARβ/δ in this process has yet to be uncovered.

Mitochondrial dysfunction is a key determinant of I/R injury and subsequent heart dysfunction. Damage to the electron transport system of cardiac mitochondria begins early during ischemia and it is a critical driver of cardiomyocyte death during reperfusion, mainly because of excessive ROS production and collapse of mitochondrial membrane potential leading to activation of apoptotic cascades (41). Thus, targeting the mitochondrial electron transport system could alleviate to some extent the mitochondrial and cellular damage that occurs in I/R. In this context, the blockade of electron transport and the partial uncoupling of respiration during ischemia or early reperfusion has been shown to decrease cardiac injury (42). In the present study, activation of PPARβ/δ enhanced FAO-dependent mitochondrial respiration and reduced ROS production in the risk area of the infarcted heart, suggesting parallel attenuation of mitochondrial oxidative damage and more efficient energy production. As a result, infarct size was reduced. Consistent with these results, a previous study has demonstrated PPARβ/δ-mediated increase in citrate synthase activity, a marker of mitochondrial oxidative metabolism, and preservation of ATP levels post- I/R (24).

Both mitochondria metabolic status and OXPHOS performance control ROS production during I/R (42). Dysregulated FA oxidative metabolism during ischemia is largely responsible for driving ROS production during reperfusion (43, 44). Decreased CPT-2 activity in the ischemic myocardium, has been associated with accumulated acyl-carnitine products and higher reperfusion damage (44). On the contrary, targeting the impaired fatty acid metabolism in peroxisomes (45) or decreasing intramitochondrial lipid accumulation (44) has been shown to be cardioprotective in I/R. In this context, upregulation of CPT-1b and CPT-2 as well as ETFDH, following PPARβ/δ activation suggests enhancement of FAO rate and modulation of the FAO-dependent electron availability, ultimately resulting in higher channeling of the available lipid substrates *via* beta-oxidation towards the ETS, through ETFDH, for energy production.

PGC-1α/NRF-1 signaling plays a vital role in cardiac myocytes. Mitochondrial respiratory capacity and biogenesis are at least in part regulated by PGC-1α-mediated activation of nuclear respiratory factor NRF-1 (46). In addition, the formation of transcriptional complexes between PGC-1α and NRF-1 promotes mitochondrial DNA transcription as well as the expression of subunits of respiratory complexes, while loss of function of either one of those factors is accompanied by defects in mitochondrial biogenesis and significant impairment of OXPHOS function (46, 47). Upregulation of PGC-1α/NRF-1 axis as well as of the downstream target SDHA after I/R in PPARβ/δ treated hearts implicates PPARβ/δ in the regulation of mitochondrial respiratory capacity and homeostasis. Taken together, the above findings suggest that the receptor stimulates both mitochondrial FAO-dependent energy metabolism and mitochondrial respiratory function to improve bioenergetics during I/R.

**Figure 7:**
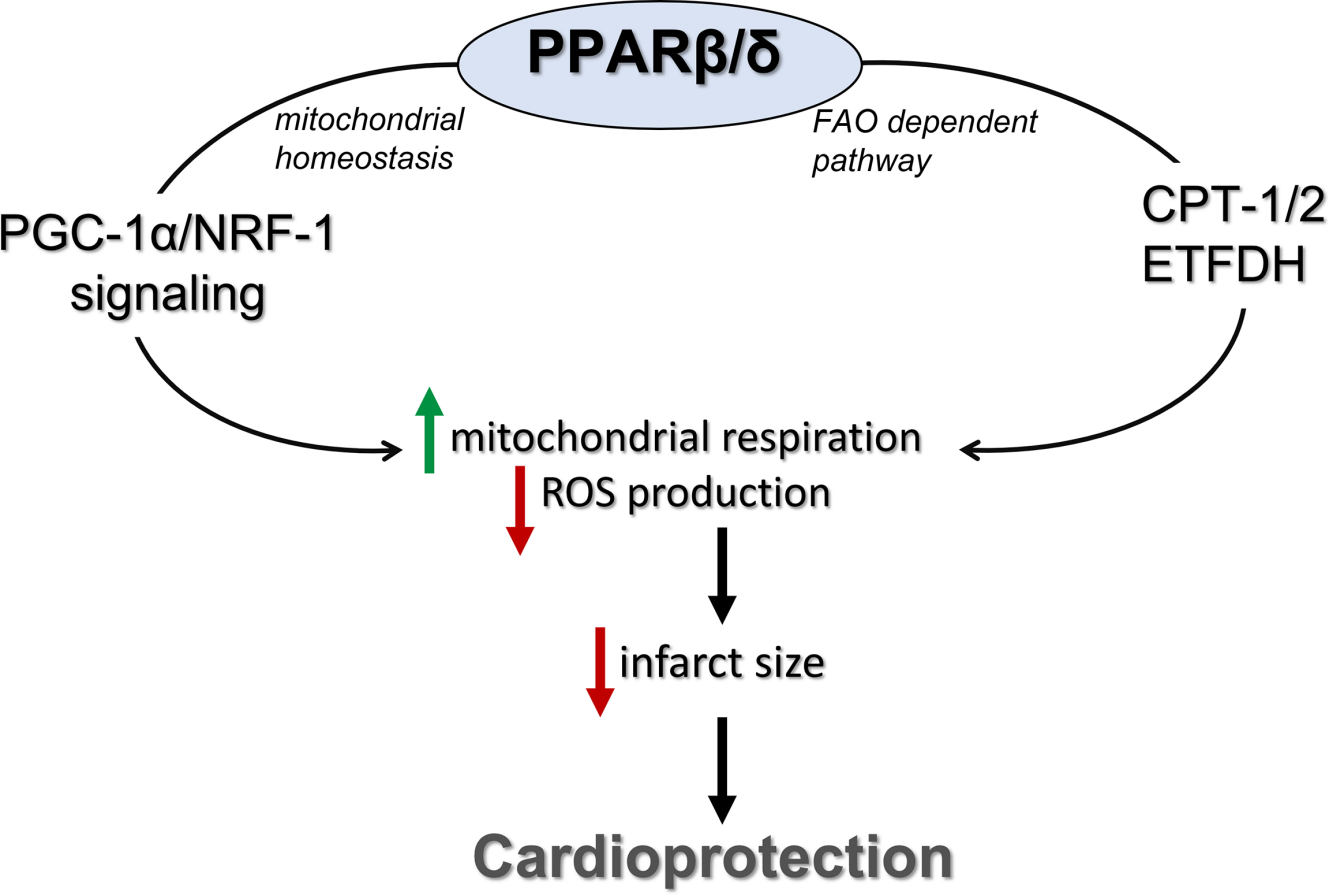
PPARβ/δ activation ameliorates mitochondrial bioenergetics during myocardial I/R through stimulation of the FA electron flow and PGC-1α/NRF-1 signaling. PPARβ/δ, peroxisome proliferator activated receptor β/δ; FAO, fatty acid oxidation; CPT-1/2, carnitine palmitoyl transferases 1 and 2; ETFDH, electron transfer flavoprotein dehydrogenase; NRF-1, nuclear respiratory factor 1; PGC-1α, PPAR gamma co-activator 1 alpha.

In conclusion, activation of PPARβ/δ leads to improvement of mitochondrial respiratory function during I/R and cardioprotection. These effects are also associated with enhanced FAO-linked respiration and PGC-1α/NRF-1 signaling (**Figure 7**). Our findings support a novel role for PPARβ/δ as a modulator of mitochondrial respiration, implicate new mechanisms that may potentially underly its cardioprotective properties and highlight its role as a potential therapeutic target for the effective management of I/R injury.

## Study limitations

The present work is limited to conclusions based on data from ex vivo experiments and thus, *in vivo* approach would be required to further support these conclusions. In addition, pharmacological intervention was given before ischemia whereas pharmacological intervention which can effectively alleviate reperfusion injury when given after cardiac ischemia is more applicable to clinical setting (5). Nevertheless, the goal of this study was to gain further mechanistic insights of the function of PPARβ/δ, a transcriptional regulator, in I/R. Given the limited time window for protective interventions during reperfusion, pretreatment with the agonist would allow adequate time for effective transcriptional activation of PPARβ/δ target genes. Furthermore, the study was performed only on subsarcolemmal mitochondria. Although, interfibrillar mitochondria are more important for energy production for contraction (48), subsarcolemmal mitochondria have been demonstrated to produce larger quantities of ROS and are more prone to damage during I/R (49). As cardioprotection often manifests differently in the different mitochondrial subpopulations (48), future studies will determine the PPARβ/δ mediated effect on other mitochondria subpopulations. Lastly, despite the sample size being relatively small in some cases, statistical significance was reached. In addition, the consistency among the data presented confirms the role of PPARβ/δ activation in myocardial mitochondrial respiration after I/R.

## Data availability statement

The original contributions presented in the study are included in the article/supplementary material. Further inquiries can be directed to the corresponding author.

## Ethics statement

The animal study was reviewed and approved by Latvian Animal Protection Ethical Committee of the Food and Veterinary Service, Riga, Latvia.

## Author contributions

Conceptualization, AL. Methodology, IP, MM-K, JK, and EL. Data analysis, AL, IP, MM-K, JK, and EL. Writing—original draft preparation, AL, IP, and MM-K. Writing— review and editing, AL and MD. Supervision, AL and MD. Funding, AL and MD. All authors contributed to the article and approved the submitted version.

## Funding

The work was supported by core institutional funds and the Graduate Program “Applications of Biology” of the School of Biology, Aristotle University of Thessaloniki.

## Acknowledgments

The authors thank Dr Reinis Vilskersts for his assistance during *ex vivo* experimental procedures and Stanislava Korzh for her assistance during high resolution fluorespirometry procedures. This article is based upon work from COST Action EU-CARDIOPROTECTION CA16225 supported by COST (European Cooperation in Science and Technology).

## Conflict of interest

The authors declare that the research was conducted in the absence of any commercial or financial relationships that could be construed as a potential conflict of interest.

## Publisher’s note

All claims expressed in this article are solely those of the authors and do not necessarily represent those of their affiliated organizations, or those of the publisher, the editors and the reviewers. Any product that may be evaluated in this article, or claim that may be made by its manufacturer, is not guaranteed or endorsed by the publisher.
